# Layer-specific strain in patients with cardiac amyloidosis using tissue tracking MR

**DOI:** 10.3389/fradi.2023.1115527

**Published:** 2023-08-03

**Authors:** Zheng Li, Cheng Yan, Guo-Xiang Hu, Rui Zhao, Hang Jin, Hong Yun, Zheng Wei, Cui-Zhen Pan, Xian-Hong Shu, Meng-Su Zeng

**Affiliations:** ^1^ Department of Echocardiography, Zhongshan Hospital, Fudan University, Shanghai, China; ^2^ Shanghai Institute of Cardiovascular Disease, Shanghai, China; ^3^ Shanghai Institute of Medical Imaging, Shanghai, China; ^4^ Department of Radiology, Zhongshan Hospital, Fudan University, Shanghai, China; ^5^ Department of Medicine, John H. Stroger, Jr. Hospital of Cook County, Chicago, IL, United States; ^6^ Department of Hematology, Zhongshan Hospital, Fudan University, Shanghai, China

**Keywords:** amyloidosis, magnetic resonance imaging, left ventricular function, layer specific strain, late gadolinium enhanced (LGE)

## Abstract

**Background:**

Cardiac infiltration is the major predictor of poor prognosis in patients with systemic amyloidosis, thus it becomes of great importance to evaluate cardiac involvement.

**Purpose:**

We aimed to evaluate left ventricular myocardial deformation alteration in patients with cardiac amyloidosis (CA) using layer-specific tissue tracking MR.

**Material and Methods:**

Thirty-nine patients with CA were enrolled. Thirty-nine normal controls were also recruited. Layer-specific tissue tracking analysis was done based on cine MR images.

**Results:**

Compared with the control group, a significant reduction in LV whole layer strain values (GLS, GCS, and GRS) and layer-specific strain values was found in patients with CA (all *P* < 0.01). In addition, GRS and GLS, as well as subendocardial and subepicardial GLS, GRS, and GCS, were all diminished in patients with CA and reduced LVEF, when compared to those with preserved or mid-range LVEF (all *P* < 0.05). GCS showed the largest AUC (0.9952, *P *= 0.0001) with a sensitivity of 93.1% and specificity of 90% to predict reduced LVEF (<40%). Moreover, GCS was the only independent predictor of LV systolic dysfunction (Odds Ratio: 3.30, 95% CI:1.341–8.12, and *P *= 0.009).

**Conclusion:**

Layer-specific tissue tracking MR could be a useful method to assess left ventricular myocardial deformation in patients with CA.

## Introduction

Systemic amyloidosis can lead to a progressive accumulation of insoluble amyloid protein fibrils in multiple organs, thus destroying the normal tissue structure and function (1–5). Cardiac amyloidosis (CA) is a major cause of mortality in patients with amyloidosis because it leads to heart failure and lethal arrhythmia (6, 7). Thus, early detection of cardiac involvement and an evaluation of heart dysfunction are important determinants in patients’ prognosis (8).

With the unique capacity for non-invasive analysis of the heart structure and function, echocardiography has become the conventional diagnostic method for many kinds of cardiovascular diseases, including CA. Speckle-tracking echocardiography-derived global longitudinal strain (GLS) provides an alternate measure for systolic dysfunction, which has been shown to be superior to left ventricular (LV) ejection fraction (EF) in identifying various cardiovascular diseases (9).

Recently, tissue tracking MR based on cine-imaging was introduced as a novel method to evaluate LV deformation by tracking the whole myocardial tissue voxel motion on routine MR cine images (6, 10, 11). There is also good agreement in strain measurements between CMR and echocardiography (6). Additionally, myocardial orientations in LV myocardial layers are heterogeneous, and there is a gradient in the myocardial deformation across the LV wall (9). Previous studies illustrated layer-specific strain analysis the potential for the diagnostic performance of ischemic heart diseases and non-ischemic heart diseases (9, 12). However, no study has reported layer-specific strain measurement alterations in patients with CA.

Accordingly, the purposes of our study were to: (1) assess the alteration of global strains at different layers of LV myocardia in patients with CA using layer-specific tissue tracking MR; (2) evaluate the power of different layer-specific LV deformation parameters to discriminate CA patients with reduced systolic function (LVEF < 40%) from those with mid-range (40% ≤ LVEF < 50%) or preserved LVEF (≥50%).

## Material and methods

### Study population

The present study enrolled 57 consecutive patients with suspected CA who were referred to the Department of Radiology, Zhongshan Hospital for cardiovascular MR imaging from September 2016 to September 2018. Amyloidosis was confirmed by positive birefringence with Congo red staining under polarized light for the biopsy of at least one involved organ. CA was evidenced by end-diastolic LV wall thickness >12 mm without any other identified cause and a diffuse pattern of enhancement on LGE imaging (1, 2, 13). The exclusion criteria for our study included contraindications for CMR examination (e.g., GFR <30 ml/min/1.73 m^2^) (1, 2, 15), diseases with increased cardiac afterload (e.g., hypertension and mid-severe aortic valvular stenosis), severe coronary artery disease, congenital heart disease, incomplete MR examinations, and poor image quality. In total, 39 patients were recruited for the analysis. We also randomly enrolled 39 healthy volunteers with no history or risk factors of heart disease as healthy controls. This study was approved by the institutional review board committee, and all patients provided written informed consent.

### CMR imaging protocol

CMR imaging was performed on a clinical 1.5 Tesla whole-body scanner (Magnetom Aera; Siemens Healthcare, Erlangen, Germany), using a spine and 18-channel body phased-array coil during a breath hold. End expiratory cine images were obtained including consecutive short-axis covering the whole LV and standard long axis (two-, three-, and four-chamber views) (2, 15). Parameters were as follows: repetition time (TR): 35.5 ms; echo time (TE): 1.1 ms; slice thickness: 8 mm; flip angle: 60°. LGE images (field of view: 340 mm × 329 mm, TR: 740 ms, TE: 3 ms, slice thickness: 8 mm, flip angle: 25°, and inversion time: 300 ms) were all acquired in imaging planes matched to cine images 10 min. after intravenous injection of contrast agent (Magnevist, Bayer Healthcare, Berlin, Germany). All participants were stable during the entire examination period.

### Image analysis

Cardiovascular MR image analysis was performed by two experienced cardiovascular radiologists who were independent and blind to the subjects’ clinical information.

Body surface area indexed LV end-diastolic volume (LVEDVi), LV end-systolic volume (LVESVi), LV stroke volume (SVi), and LVEF were assessed using dedicated software (Argus, Siemens Healthcare). Papillary muscles and trabeculations were excluded from myocardial mass and recorded in LV volume.

Endo- and epicardial borders of LV were manually drawn at the end-diastolic phase of all cine images using off-line commercial software (CVI 42, vs. 5.2.2; Circle Cardiovascular Imaging, Calgary, Alberta, Canada) by the two cardiovascular radiologists mentioned above. The insertion of the right ventricle and LV at the end-diastolic phase of short-axis images were defined as short-axis reference points, and the rest phases of all cine images were automatically traced and segmented according to the 16 American Heart Association segmentation of a bull’s-eye plot. LV global whole layer strain values were automatically calculated in longitudinal, circumferential, and radial directions, then expressed as GLS, global circumferential strain (GCS), and global radial strain (GRS), as described in the study of Xu et al. (9). Moreover, peak longitudinal strain from all long-axis slices was averaged to provide subendocardial GLS (GLSendo) and subepicardial GLS (GLSepi); similarly, peak circumferential strain from all short-axis slices were averaged to provide subendocardial GCS (GCSendo) and subepicardial GCS (GCSepi). In addition, subendocardial GRS (GRSendo) and subepicardial GRS (GRSepi) were recorded as the average systolic strain from all slices. Moreover, the relative subendocardial-subepicardial strain value gradients were defined were defined as subendocardial 22 strain value minus subepicardial strain relative to subendocardial strain.

$$\%\Delta GLS=\frac{GLSendo-GLSepi}{GLSendo},$$

$$\%\Delta GCS=\frac{GCSendo-GCSepi}{GCSendo},$$

$$and\;\%\Delta GRS=\frac{GRSendo-GRSepi}{GRSendo}.$$

### Subgroup analysis

A sub-analysis was performed to evaluate the differences in layer-specific parameters stratified by LVEF. Patients with CA who had preserved LVEF (≥50%) or mid-range LVEF (40% ≤ LVEF < 50%) were compared to those with a reduced LVEF (<40%).

### Intra- and interobserver variabilities

Intra- and interobserver variabilities for strain values were assessed in 15 randomly selected patients with CA using intraclass correlation coefficient (ICC) analysis. Interobserver variability was assessed on the same image set analyzed by two independent cardiovascular radiologists. Intraobserver variability was assessed on the same image set by one cardiovascular radiologist 2 weeks later.

### Statistical analyses

All statistical analyses were performed using IBM SPSS Statistics version 25 (IBM Corp., Armonk, NY, USA) and GraphPad Prism version 8.0.0 (GraphPad Software Inc., San Diego, CA, USA). Continuous variables were tested for normality using the Kolmogorov-Smirnov normality test and were presented as mean ± standard deviation, while categorical variables were expressed as counts with percentages. Differences in continuous data were compared using a student t-test. Categorical variables were compared by χ^2^ or Fisher exact tests (two-sided). Correlations were assessed by Pearson correlation or Spearman coefficient. The diagnostic accuracy of LV deformation parameters was evaluated by means of area under the curve (AUC) of receiver operating characteristic (ROC) and logistic regression; odds ratio(OR) and 95% CIs were calculated. A two-sided *P*-value < 0.05 was considered statistically significant.

## Results

### Baseline characteristics

Baseline clinical, echocardiography, and CMR characteristics are summarized in Table 1. There were 23 men and 16 women (mean age 49.15 ± 13.86 years) in the control group. Compared with the control group, there was a significantly increased heart rate, left atrial dimension, LV end-systolic dimension, interventricular septum thickness, LV post-wall thickness, pulmonary arterial systolic pressure (PASP), and LVESVi, whereas there was a significant decrease in SVi in patients with CA. No significant difference was found in gender, age, LV end-diastolic dimension, or LVEDVi (*P* > 0.05, Table 1).

**Table 1:** Clinical, echocardiographic, and cardiac magnetic resonance imaging characteristics for all groups.

Parameters	Control group (*n* = 39)	CA	*P1*	CArEF (*n* = 29)	CApEF (*n* = 10)	*P2*
Clinical characteristics						
Age (years)	49.15 ± 13.86	54.69 ± 12.25	0.061	53.55 ± 12.31	58.40 ± 11.92	0.286
Gender, Male [*n* (%)]	23 (58.97%)	30 (76.92%)	0.072	22 (75.86%)	8 (80.00%)	0.581
Hemodynamic data						
Heart Rate	69.00 ± 12.26	80.77 ± 14.60	0.001	82.21 ± 15.61	76.60 ± 10.76	0.301
Echocardiology						
LAD (mm)	34.38 ± 4.18	45.00 ± 7.25	0.000	44.93 ± 8.11	45.22 ± 3.80	0.918
LVEDD (mm)	45.73 ± 4.75	45.05 ± 9.04	0.701	45.82 ± 9.47	42.67 ± 7.52	0.370
LVESD (mm)	29.23 ± 2.83	32.97 ± 7.99	0.012	34.36 ± 8.28	28.67 ± 5.32	0.062
IVST (mm)	8.96 ± 1.40	14.27 ± 2.22	0.000	14.04 ± 2.32	15.00 ± 1.80	0.263
PWT (mm)	8.58 ± 1.14	13.35 ± 2.52	0.000	13.07 ± 2.51	14.22 ± 2.49	0.238
PASP (mmHg)	31.54 ± 3.61	40.89 ± 10.98	0.000	42.36 ± 11.90	36.33 ± 5.77	0.155
CMR parameters						
LVEDVi (ml/m^2^)	73.93 ± 15.78	80.41 ± 32.19	0.268	84.22 ± 34.68	69.35 ± 21.25	0.212
LVESVi (ml/m^2^)	30.89 ± 9.16	52.29 ± 28.78	0.000	58.19 ± 30.70	35.16 ± 11.23	0.002
SVi (ml/m^2^)	43.08 ± 8.37	28.12 ± 9.63	0.000	26.03 ± 7.81	34.19 ± 12.12	0.019
LVEF (%)	59.68 ± 6.38	37.48 ± 10.55	0.000	32.87 ± 7.46	50.87 ± 5.85	0.000

CA, cardiac amyloidosis; LAD, left atrial dimension; LVEDD, left ventricular end-diastolic dimension; LVESD, left ventricular end-systolic dimension; IVST, interventricular septum thickness; PWT, left ventricular post wall thickness; PASP, pulmonary arterial systolic pressure; LVEDVi, body surface area indexed left ventricular end-diastolic volume; LVESVi, body surface area indexed left ventricular end-systolic volume; Svi, body surface area indexed systolic volume; LVEF, left ventricular ejection fraction.

P1: CA vs. control group; P2: CArEF vs. CApEF.

### LV global deformation parameters

**Figure 1:**
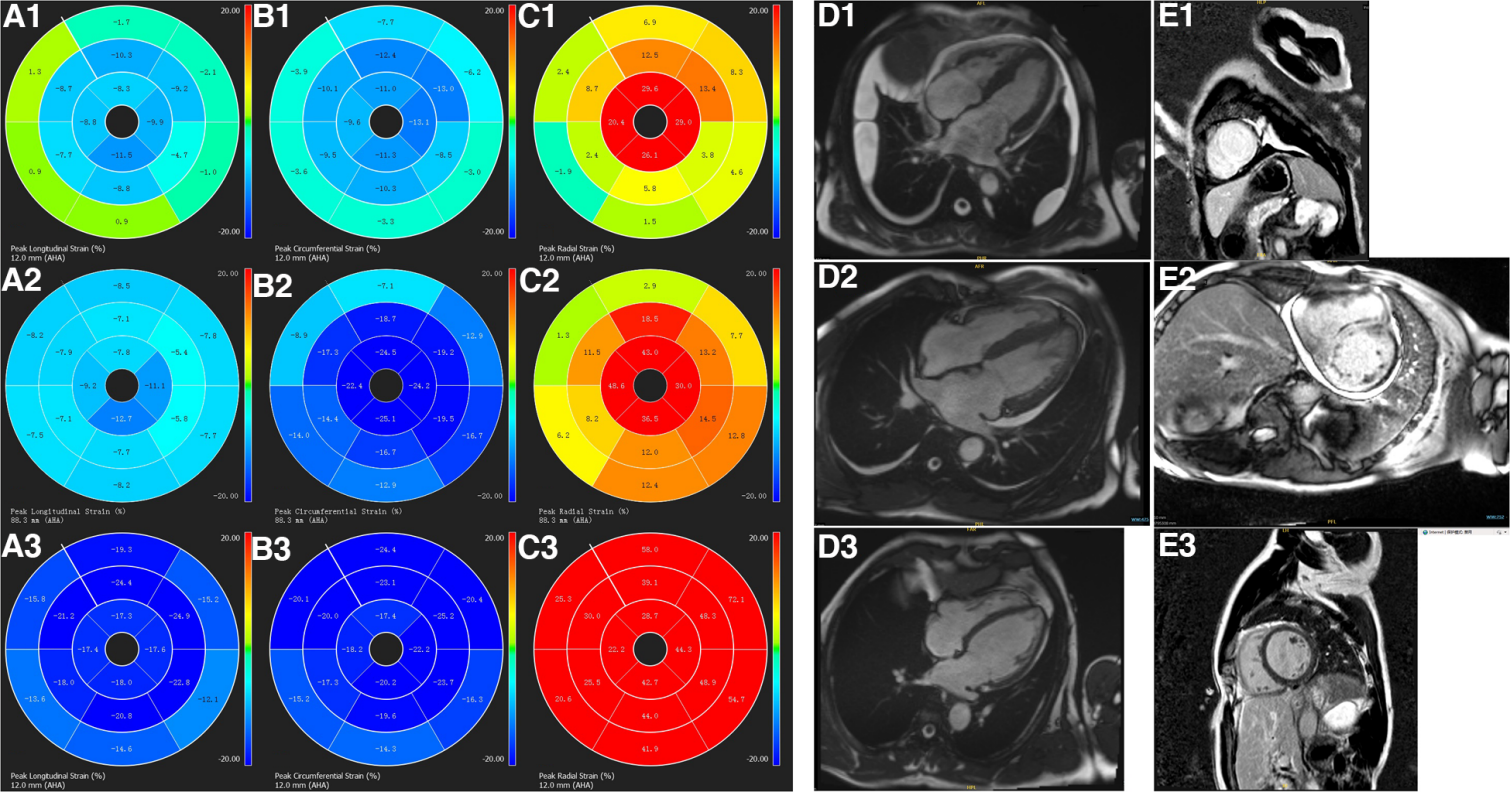
The characterization of left ventricular strain polar maps, cine imaging, and LGE imaging. Longitudinal (panels **A1**–**A3**), circumferential (panels **B1**–**B3**), radial strain polar maps (panels **C1**–**C3**), cine imaging (panels **D1**–**D3**), and LGE imaging (panels **E1**–**E3**). A healthy volunteer has normal cine (**D3**), LGE images (**E3**), normal left ventricular longitudinal (**A3**), circumferential (**B3**), and radial strain (**C3**) values. While a patient with CA and preserved LVEF (**D2**) has left ventricular transmural enhancement on LGE imaging (**E2**), reduced longitudinal (**A2**), circumferential (**B2**), and radial (**C2**) strain values. A patient with CA and reduced LVEF (**D1**) also has transmural enhancement on left ventricular LGE imaging (**E1**) and even reduced longitudinal (**A1**), circumferential (**B1**), and radial (**C1**) strain values.

Table 2 summarizes the LV deformation analysis findings. A significant reduction in LV strain values, including whole layer and layer-specific components, was found in patients with CA when compared to the control group. These findings suggest LV deformation deterioration in patients with CA. (Table 2; Figure 1).

**Table 2:** Left ventricular deformation parameters for all groups.

LV strain variables (%)	Control group (*n* = 39)	CA (*n* = 39)	*P1*	CArEF (*n* = 29)	CApEF (*n* = 10)	*P2*
GLS	−15.14 ± 12.71	−6.49 ± 3.12	0.000	−5.82 ± 2.42	−8.44 ± 4.15	0.085
GLSendo	−17.15 ± 1.62	−7.41 ± 2.79	0.000	−6.78 ± 2.17	−9.24 ± 3.62	0.014
GLSepi	−16.48 ± .1.60	−7.14 ± 2.58	0.000	−6.47 ± 2.01	−9.08 ± 3.16	0.004
%△GLS	3.86 ± 3.82	1.99 ± 12.57	0.381	4.12 ± 8.90	−4.16 ± 19.09	0.245
GCS	−18.74 ± 2.47	−10.89 ± 3.64	0.000	−9.47 ± 2.96	−15.01 ± 1.81	0.000
GCSendo	−20.92 ± 2.23	−12.91 ± 3.75	0.000	−11.57 ± 3.25	−16.78 ± 2.07	0.000
GCSepi	−16.74 ± 1.73	−8.46 ± 2.29	0.000	−7.71 ± 2.03	−10.67 ± 1.42	0.000
%△GCS	19.33 ± 4.63	33.44 ± 7.37	0.000	32.46 ± 7.81	36.28 ± 5.26	0.160
GRS	31.92 ± 7.57	13.74 ± 6.50	0.000	11.37 ± 4.68	20.61 ± 6.27	0.000
GRSendo	36.33 ± 6.08	17.45 ± 6.49	0.000	15.00 ± 5.09	24.57 ± 4.63	0.000
GRSepi	27.16 ± 3.68	10.61 ± 3.41	0.000	9.41 ± 2.76	14.10 ± 2.73	0.000
%△GRS	24.11 ± 6.42	37.32 ± 9.12	0.000	35.65 ± 9.16	42.17 ± 7.47	0.050

GLS, left ventricular global longitudinal strain; GCS, left ventricular global circumferential strain; GRS, left ventricular global radial strain; GLSendo, endocardial GLS; epicardial GLSepi, epicardial GLS; %△GLS, relative differences of endocardial and eipicaridal GLS; GCSendo, endocardial GCS; GCSepi, epicardial GCS; %△GCS, relative differences of endocardial and eipicaridal GCS; GRSendo, endocardial GRS; GRSepi, epicardial; %△GRS, relative differences of endocardial and eipicaridal GRS.

Other abbreviations are as those in Table 1.

*P*1: CA vs. the control group and *P*2: CArEF vs. CApEF.

### Subgroup analysis

Of all patients with CA, 10 (eight men and two women, mean age 58.40 ± 11.92 years; age range 35–72 years) had preserved (≥50%) or mid-range LVEF (40% ≤ LVEF < 50%) and 29 (21 men and 8 women, mean age 53.55 ± 12.31 years; age range 18–78 years) did not (LVEF < 40%). There were significantly increased LVESVi and decreased SVi in patients in the CArEF group (*P* < 0.05, Table 1)when compared to those with CApEF. No statistically significant difference was found with regard to other measures of echocardiography or cardiac MR volume parameters.

When compared to the CApEF group, GRS and GCS were diminished in patients in the CArEF group, whereas no significant difference in GLS was found between the two subgroups. The comparison of layer-specific LV deformation parameters also showed notable findings; subendocardial and subepicardial components of GLS, GRS, and GCS were all reduced in patients in the CArEF group when compared to the CApEF group (Tables 1,2, and Figure 1).

### The value of strain parameters as predictors of reduced LVEF

**Figure 2:**
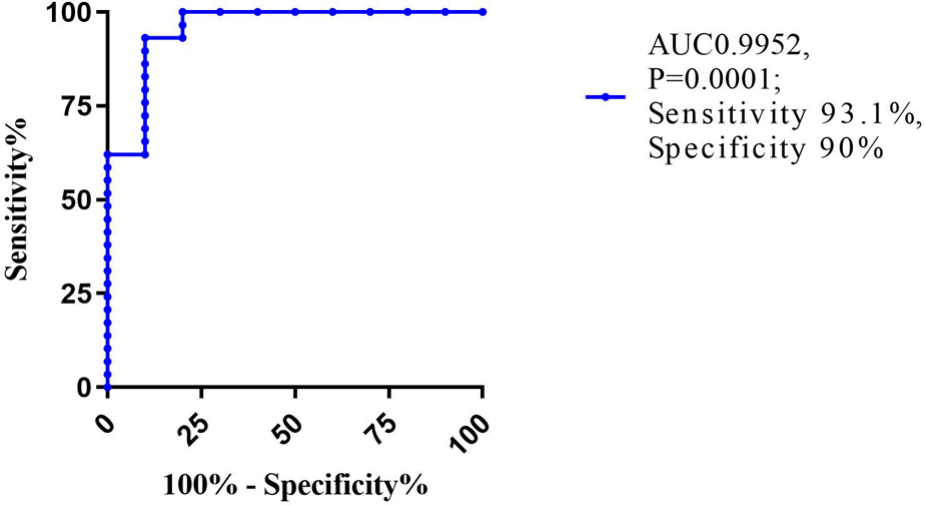
Receiver operating characteristic (ROC) curve for GCS. AUC, area of under curve.

When reduced LVEF (<40%) was used as a dependent variable, the logistic regression model showed that GCS was an independent predictor of LVEF reduction (OR: 3.30, 95% CI:1.341–8.12, and *P *= 0.009). ROC curve analyses demonstrated that GCS showed the largest AUC (0.9952, *P *= 0.0001) with a sensitivity of 93.1% and specificity of 90% to predict reduced LVEF (Figure 2).

### Intra- and interobserver variability

As shown in Table 3, the inter- and intraobserver variability of all LV deformation parameters was calculated. The ICC values showed moderate to excellent reproducibility of all LV deformation parameters (intraobserver ICC ≥ 0.892, *P* all <0.01; interobserver ICC ≥0.731, *P* all <0.05).

**Table 3:** Inter- and intraobserver variability of left ventricular deformation parameters by ICC analysis.

	Intraobserver			Interobserver		
	ICC	95% CI	*P*	ICC	95% CI	*P*
GLS	0.959	0.883–0.986	0.000	0.924	0.789–0.974	0.000
GLSendo	0.987	0.946–0.997	0.000	0.983	0.932–9.996	0.000
GLSepi	0.985	0.940–0.996	0.000	0.980	0.919–0.995	0.000
%△GLS	0.892	0.566–0.973	0.001	0.731	−0.085–0.933	0.032
GCS	0.967	0.905–0.989	0.000	0.961	0.887–0.987	0.000
GCSendo	0.988	0.951–0.997	0.000	0.995	0.979–0.999	0000
GCSepi	0.986	0.942–0.996	0.000	0.988	0.950–0.997	0.000
%△GCS	0.936	0.744–0.984	0.000	0.850	0.396–0.963	0.000
GRS	0.977	0.932–0.992	0.000	0.952	0.863–0.984	0.000
GRSendo	0.996	0.986–0.999	0.000	0.993	0.972–0.998	0.000
GRSepi	0.989	0.955–0.997	0.000	0.996	0.985–0.999	0.000
%△GRS	0.926	0.703–0.982	0.000	0.922	0.686–0.981	0.000

ICC, intraclass correlation coefficient, other abbreviations as those in Tables 1,2.

## Discussion

### Main findings

In this study, we assessed LV deformation in patients with CA using layer-specific tissue tracking MR, and our study yielded several important findings. First, whole-layer and layer-specific LV deformation parameters, including GLS, GCS, and GRS, were all significantly diminished in patients with CA. Second, when compared to the patients with preserved or mid-range LVEF, GRS and GLS, as well as subendocardial and subepicardial GLS, GRS, and GCS, were all incrementally diminished in patients with reduced LVEF. Last, GCS was the only independent predictor of LVEF reduction to below 40% in patients with CA.

### Layer-specific tissue tracking MRI

Heart involvement is a strong predictor of poor outcomes in patients with systemic amyloidosis (16, 17); the etiology of cardiac dysfunction in patients with CA might include: (1) restriction of the myocardia due to the infiltration of amyloid fibrils into the myocardial interstitial matrix, (2) myocardial edema induced by specific cardiotoxic effects of amyloidosis precursor in the circulation, (3) microvascular ischemia-induced cellular ischemia and metabolic dysfunction, and/or (4) secondary myocardial fibrosis (13, 16, 18–22). Although myocardial biopsy is considered the gold standard for assessing heart involvement in patients with CA, due to the invasive nature of associated potential complications, diagnoses of CA are usually made by serum cardiac biomarkers, electrocardiography (ECG), echocardiography, and other cardiac imaging methods in clinical practice (16, 23).

Cardiovascular MR has emerged as an effective non-invasive diagnostic technique for patients with CA. The characteristic manifestation and extent of left ventricular (LV) diffused transmural late gadolinium enhancement (LGE) is associated with the burden of interstitial fibril protein infiltration and myocardial viability (24–31). But the use of contrast medium is often restricted in patients with suspected CA who also have significant renal function impairment, and some studies reported that LGE cannot absolutely quantify diffuse myocardial fibrosis (26, 32, 33). Recently, considerable interest has emerged in using tissue tracking MR to quantitatively evaluate LV global and segmental myocardial deformation (2).

Layer-specific strain measurements provide better detection of subtle myocardial pathology (34). To the best of our knowledge, this is the first study to investigate the differences in layer-specific strain measurements in patients with CA. Previous studies proved that GLS was sensitive in predicting subclinical LV contractile dysfunction on early-stage heart failure (HF) (2, 35), and reduced GLS was associated with a shortened survival of patients with CA (3, 18, 36). Wan et al. (2) found patients with CA had reduced GLS, GCS, and GRS, compared with healthy controls and patients without clinical CA. In accordance with previous studies, our study revealed a significant degradation of whole layer and layer-specific GLS, as well as GRS and GCS, in CA patients, compared with healthy volunteers. Moreover, besides decreased subendocardial and subepicardial strain measurements, our study also documented incrementally increased relative gradient in GRS and GCS of patients with CA, when compared to healthy volunteers, whereas no significant difference in %△GLS was found between patients with CA and healthy volunteers. Therefore, our results may indicate that tissue tracking MR imaging-derived LV deformation parameters could be a simple method to detect LV myocardial systolic function in patients with CA. Although the physiologic underpinning of more severely deteriorated subepicardial layer strain in patients with CA is unclear, we speculate that these results may reflect LV tissue characteristics of amyloid fibers infiltration and structural remodeling (9).

### Strain measurements in LV systolic function stratification

Myocardial infiltration of amyloid fibrils typically leads to restrictive cardiomyopathy, then progressive congestive heart failure (HF), and even sudden death. Survival time after diagnosis could shorten significantly if patients presented with congestive HF (7). Consequently, the evaluation of heart function and early intervention of specific management are also important for patients’ survival. Setting 40% of LVEF as the cutoff point of LV systolic function, our study evaluated the ability of strain measurements to predict LV systolic dysfunction in patients with CA, and our study demonstrated that strain measurements, including whole-layer GRS, GCS, and the layer-specific GLS, GRS, and GCS were found incrementally decreased in patients whose LVEF fell to below 40%; in addition, ROC curve analysis and logistic regression analysis also illustrated that GCS was the independent factor of predicting LV systolic dysfunction with high sensitivity and specificity. These results suggest that LV strain measurements may provide detailed information on the systolic function and may serve as an alternative method for detecting LV systolic dysfunction in patients with CA.

Interestingly, the discrepancy of GLS, GCS, and GRS and their layer-specific components to predict LV systolic dysfunction was found in the present study. In the study of Xu et al. (9), they found that GLSepi, GRS, as well as the difference in endocardial and epicardial strains were sensitive to systolic dysfunction among HF patients with preserved LVEF. Stokke et al. (36) performed a combined mathematical and echocardiographic study, and they found that GCS contributes more than twice as much to EF than GLS. Thus, the discrepancy of strain measurements in the study can be explained that GLS may be more sensitive in systolic function, which affects the subendocardial region first. However, both GLS and GCS deterioration may suggest a more transmural dysfunction affecting circumferential fibers in the mid-layer (36). In consistency with the theory, the CA patients enrolled in our study were all confirmed with LV transmural LGE, which is suggestive that cardiac infiltration of amyloid fibril may have been expanded to the mid and even all cardiac layers. The relative increase in GCS and GRS in patients with LVEF reserve than in patients with reduced LVEF is also in accordance with the theory that mid-layer myocardial fibers compensate for the loss of longitudinal mechanic to preserve LV pump function (36).

### Limitations

There were several limitations that warrant comments on our study. First, we used combined criteria with LV wall thickness, LV transmural enhancement on LGE, and biopsy from any site as the definition of cardiac amyloidosis, thus myocardial biopsy was not done in the present study. However, this combined criterion was reported to be sensitive and specific for CA. Second, the sample size was relatively small; we recruited CA patients with transmural LGE, but the LGE pattern may be atypical and patchy in patients with CA. Thus, some patients may be excluded from our study. Third, other diseases causing LV wall thickening were not included in this research, such as hypertensive heart disease and hypertrophic cardiomyopathy. Finally, this was a cross-sectional study without long-term follow-up. For these reasons, further investigations with larger numbers of different types of patients are required to confirm the predictive value of LV deformation parameters.

## Conclusions

In conclusion, layer-specific tissue tracking MR imaging provides a useful non-invasive method to evaluate LV systolic dysfunction in patients with CA.

## Data Availability

The raw data supporting the conclusions of this article will be made available by the authors, without undue reservation.
